# Connexins in Acquired Hearing Loss: Expanding Research Perspectives

**DOI:** 10.3390/biomedicines13123109

**Published:** 2025-12-17

**Authors:** Sihan Huang, Jingyi Zhu, Jifang Zhang, Tianyu Gong, Zhongyuan Fei, Penghui Chen, Shule Hou, Jun Yang

**Affiliations:** 1 Department of Otorhinolaryngology-Head and Neck Surgery, Xinhua Hospital, Shanghai Jiao Tong University School of Medicine, Shanghai 200082, China; 2 Ear Institute, Shanghai Jiao Tong University School of Medicine, Shanghai 200082, China; 3 Shanghai Key Laboratory of Translational Medicine on Ear and Nose Diseases, Shanghai 200082, China

**Keywords:** acquired hearing loss, age-related hearing loss, noise-induced hearing loss, gap junction, cochlea, connexin26, connexin30

## Abstract

Connexins, as key players in intercellular communication in the inner ear, are vital for maintaining normal hearing function. While numerous studies have explored their role in congenital hereditary hearing loss, the underlying mechanisms and therapeutic potential of connexins in acquired hearing loss remain to be fully elucidated. This review summarizes recent advances in connexin research in the context of acquired hearing loss, with a focus on presbycusis, noise-induced, and drug-induced hearing loss, and delves into their pathophysiological roles. Through the analysis and organization of these research findings, the article aims to provide a theoretical basis and research direction for future connexin-targeted therapies for acquired hearing loss.

## 1. Introduction

Acquired hearing loss, caused by postnatal factors like aging, infections, trauma, noise exposure, or ototoxic substances, is marked by reduced hearing sensitivity or decreased speech recognition. It can occur at any life stage but is more common in adults. This review focuses on sensorineural acquired hearing loss, which stems from inner ear or auditory nerve damage.

Gap junctions (GJs) are crucial in connecting cells within the organ of Corti (OC) and stria vascularis (SV), aiding intercellular communication necessary for maintaining proper ionic balance and auditory function. These GJs facilitate the regulation and transport of ions, electrolytes, secondary messengers, and metabolites among cochlear cells [1,2]. The intercellular channel is formed by the head-to-head contact between a connexon in one cell and another connexon in a neighboring cell. A connexon is a hexameric ring structure composed of six connexin molecules, which can form a pore within the lipid bilayer of the plasma membrane.

Based on the sequence homology of connexins, these proteins can be categorized into five subfamilies (α, β, γ, δ, ε). The predominant connexins in the cochlea are Cx26 and Cx30, both belonging to the β-connexin. Furthermore, studies have reported the expression of additional connexins (e.g., Cx29, Cx31, Cx31.9, Cx32, Cx43 and Cx45) [3,4,5,6].

**Figure 1:**
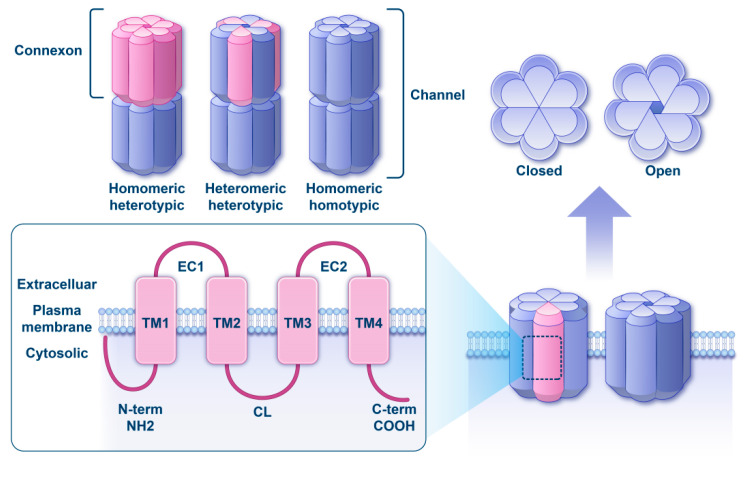
Schematic overview of connexin. (Upper half) Three functional gap-junction channel configurations are shown, distinguished by connexon composition and pairing. A homomeric heterotypic channel is formed by the docking of two different homomeric connexons. A heteromeric heterotypic channel results from the association of one heteromeric connexon with a second, compositionally distinct connexon. A homomeric homotypic channel is generated by the head-to-head joining of two identical homomeric connexons. (Right) Side-view cartoons depict the closed and open states of a connexon hemichannel. (Lower half) The basic membrane topology of connexin-26 is illustrated, highlighting four α-helical transmembrane segments (TM1–TM4), two extracellular loops (EC1 and EC2), one cytoplasmic loop (CL), and N- and C-termini that both reside on the cytosolic side.

The formation of hemichannels involves either a single connexin type (homomeric) or multiple types (heteromeric). GJ channels result from the pairing of two hemichannels; they are homotypic if the paired hemichannels are identical, and heterotypic if they differ (Figure 1) [7]. This channel composition is dictated by the particular set of connexins expressed by a given cell or tissue, along with the intrinsic ability of compatible connexins to combine [8,9]. Undocked or unopposed connexons can exist as hemichannels, allowing for the transfer of molecules between the extracellular and intracellular environments.

Cx26 is encoded by *GJB2*, while Cx30 is encoded by *GJB6*. *GJB2* and *GJB6* exhibit widespread expression profiles across two critical anatomical domains: supporting cells of the cochlear epithelium and connective matrices within the inner ear. Mutations in *GJB2* and *GJB6* cause hereditary hearing loss. The Deafness Variation Database (DVD, https://deafnessvariationdatabase.org/genes/GJB2; accessed on 24 May 2025) has cataloged 1165 distinct sequence variants within the *GJB2* locus. Of these variants, 428 (36.8%) have been designated as pathogenic or likely pathogenic. Mutations in GJ protein genes are well established as a primary cause of congenital deafness [10,11]. The mechanism behind hearing loss is thought to involve the role of GJs in K^+^ recycling [12,13]. Furthermore, research suggests that GJs may also influence intercellular Ca^2+^ signaling pathways by releasing ATP during postnatal auditory development [14,15]. Another perspective highlights the importance of the glucose transport pathway facilitated by GJs in the differentiation and maturation of supporting cells, particularly inner and outer column cells of the tunnel of Corti in early development [16,17,18]. However, none of these hypotheses fully elucidate the intricate mechanisms involved in the pathogenesis of Cx26-related hearing impairment. This is primarily due to the fact that hemichannels and GJ channels formed by the Cx26 protein appear to serve multiple roles in the development and function of inner ear structures.

Numerous studies have elucidated the association between connexin mutations and congenital deafness. However, recent studies have suggested a potential connection between GJs and the onset and progression of acquired hearing loss [19,20,21].

## 2. Age-Related Hearing Loss (ARHL)

ARHL, also known as presbycusis, is a common neurodegenerative and communication disorder affecting over one-third of the global population [22,23]. ARHL is a complex sensory disorder characterized by reduced sensitivity to sound, especially at higher frequencies, difficulties in speech perception-particularly in noisy environments, and slow processing of acoustic information in the central auditory system [24,25]. The onset of presbycusis is influenced by a mix of external and internal factors such as genetics, aging, medication usage, and exposure to loud noise [26,27]. According to various twin and longitudinal studies, the heritability rate of ARHL is estimated to be between 35% and 55%.

Irreversible damage to hair cells and spiral ganglion cells and the degeneration of SV are the main pathological changes in ARHL. The aging process in the cochlea leads to three primary types of changes: sensory, neural, and metabolic presbycusis [27,28]. Sensory presbycusis involves hair cell loss, neural presbycusis entails a decrease in spiral ganglion neurons (SGNs), and metabolic presbycusis is marked by the degeneration of SV. SV degeneration also leads to a decline in the endocochlear potential (EP) [29]. Current research on the underlying mechanisms of ARHL primarily centers around oxidative stress, inflammatory response, apoptosis, and cellular protection through autophagy. Despite progress in identifying aging-related cellular and molecular changes in the inner ear, the question remains: what are the specific mechanisms of age-related deterioration of cochlear structure and function?

### 2.1. Cx26 Partial Loss and Cx30 Depletion Accelerated ARHL

While the link between *GJB2* or *GJB6* variants and hearing loss is well-established, the exact role of inner ear connexins in the development of deafness remains incompletely understood. Ichimiya et al. first reported that expression of Cx26 was significantly reduced in the spiral ligament of aged mice [30]. Tajima et al. discovered a significant decrease in the GJ proteins Cx26 and Cx30 in the cochlea of C57BL/6J mice at 32 weeks compared with that at 4 weeks [20]. Previous research has shown that 32-week-old C57BL/6J mice experience high-frequency hearing loss. However, there was no significant hair cell loss or visible degeneration in the sensory epithelium at this stage. Xu et al. reported that aging in mice was associated with hypermethylation of specific CpG sites in the *GJB2* promoter, without a corresponding significant alteration in *GJB2* mRNA levels compared to young mice. From this, they inferred that the decrease in Cx26 protein was not due to lower *GJB2* transcription, but rather to reduced Cx26 protein synthesis or accelerated post-synthesis degradation [31].

In older CBA/CaJ mice, similar results were noted where Cx30 and Cx43 protein levels decreased significantly in the cochlea. Interestingly, mice treated with aldosterone, a drug known to slow ARHL progression and its biomarker changes, exhibited delayed hearing loss and better hearing compared with the untreated mice of the same age, showcasing characteristics of high Cx30 expression [19]. These findings imply that cochlear GJ degradation could play a role in the early-stage pathology of ARHL.

Therefore, it would be intriguing to investigate the impact of regulating connexin expression in the cochlea on the progression of ARHL. Previous attempts to study this by mimicking Cx26 loss in mice were unsuccessful, as total removal of *Gjb2* resulted in embryonic death due to disrupted glucose transport through the placenta [32]. In order to overcome this challenge, researchers have created different *Gjb2* point mutations and conditional knockout models, which have served as useful resources for studying its disease-causing mechanism [33]. Fetoni et al. found that the auditory performance of Cx26 cKO mice (*Gjb2*^loxP/+^Sox10-Cre) degraded more rapidly over time than that of control mice, and this decline was associated with oxidative stress in the cochlea [21]. The study revealed significant apoptosis and oxidative damage in the cochlear duct of these mice, along with reduced release of glutathione from connexin hemichannels and dysregulation of genes controlled by nuclear factor erythroid 2-related factor 2 (Nrf2), a key factor in cellular response to oxidative stress [21]. Xu et al. established two conditional knockout models to reduce Cx26 expression in supporting cells of adult mice (Cx26^flox/flox^ mice with Sox2CreERT2 or Fgfr3iCreERT2), simulating the decreased Cx26 levels seen in older mice. Both models showed progressive high-frequency hearing loss over time, as well as degeneration of outer hair cells (OHCs) and SGNs, which indicates that the loss of Cx26 in the cochlea speeds up the development of ARHL. Similar audiological outcomes were observed in *Gjb2*^loxP/loxP^Rosa26creER mice (tamoxifen injected at P5 and later), p.V37I homozygous mutant mice and Cx26^+/−^ mice [34,35,36]. These mice exhibited normal hearing function and cochlear development at P30, but displayed progressive hearing loss with age in comparison with control mice.

Based on these findings, whether Cx30 deletion could increase cochlear susceptibility to aging processes and whether *Gjb6* deletion can be considered a genetic risk factor for ARHL has also attracted the attention of researchers. However, the inactivation of Cx30 results in profound deafness and a significant decrease in Cx26 expression. Restoring Cx26 expression in Cx30^−/−^ mice can reverse the congenital hearing loss, making it challenging to assess the isolated role of Cx30. This issue was partially addressed with Cx30^Δ/Δ^, a model where Cx30 was removed without disrupting surrounding sequences, enabling its successful use in basic research [37]. Notably, the deletion of Cx30 did not impact ABR thresholds in both young and adult mice, suggesting that Cx30 may not be essential for hearing. Paciello et al. were the first to discover that the absence of Cx30 can increase susceptibility to age-related processes in cochlear structures, worsening hearing loss and cellular damage in HCs and SGNs [38]. They suggested that the lack of *Gjb6* could further exacerbate ARHL, making cochlear structures more vulnerable to oxidative stress, inflammation, and vascular damage. Additionally, changes in Cx26 expression levels in the cochlea of Cx30Δ/Δ mice at 12 months of age were noted, indicating a significant decrease. The exacerbation of ARHL may be attributed to the combined effect of Cx30 and Cx26. Loss of Cx30 and concomitant downregulation of Cx26 is associated with an increase in the amount of reactive oxygen species (ROS) in aging Cx30^Δ/Δ^ cochlea, suggesting a role for connexin hemichannels in preventing oxidative stress during aging and possibly diffusion of antioxidant molecules to compensate for oxidation.

### 2.2. A88V Mutation in Cx30 Stops Early Onset ARHL

Despite extensive investigations into connexin-based therapies, the efficacy of Cx26/Cx30 upregulation in mitigating ARHL remains unsubstantiated in rodent models. The auditory phenotype of Cx30^A88V/A88V^ mice may provide further support for the aforementioned hypothesis. While researching the causative gene of hidrotic ectodermal dysplasia (HED, MIM 129500; also known as Clouston syndrome), an autosomal dominant disease, Lamartine et al. identified a transition in the *GJB6*, 263 (C → T), resulting in a missense mutation (A88V) [39].

Although sensorineural hearing loss has been reported in a few cases [40,41], the majority of CX30 A88V patients exhibit normal hearing. To investigate the pathogenic mechanisms of Clouston syndrome induced by the Cx30-A88V mutant, Bosen et al. established a mouse model on a CD-1 mouse background [42]. The Cx30-A88V mutant mice displayed hyperproliferative and enlarged sebaceous glands, along with mild palmoplantar hyperkeratosis. Interestingly, compared with wildtype and heterozygous mice, these mutant mice exhibited an atypical hearing profile compared with wild-type mice, showing elevated hearing thresholds in the lower frequency ranges (<10 kHz) in the apical region of the cochlea but improved hearing in the high frequency, basal region of the cochlea [42]. Lukashkina et al. further confirmed and expanded on this observation. They found that the active frequency tuning of the basilar membrane and the passive compliance of the cochlear partition were enhanced in CD-1Cx30^A88V/A88V^ mice compared with CBA/J mice, which are known for their sensitive high-frequency hearing [43]. Despite having a significantly reduced EP, CD-1Cx30^A88V/A88V^ mice maintained excellent sensitivity in their basal cochleae and normal saturating amplitudes of the cochlear microphonic [43].

Essenfelder et al. observed that cells expressing A88V Cx30 exhibited smaller junctional conductance compared to those connected by wild-type (WT) Cx30. They proposed that the lower conductance values in homotypic A88V pairs might arise from one or both of two mechanisms: (i) reduced inter-connexon affinity in the homotypic configuration, leading to inefficient channel formation; and/or (ii) altered intrinsic channel properties—such as favoring a closed state in the absence of a transjunctional potential—which reduces open time probability and/or unitary conductance [44]. Based on the above findings, Levic et al. demonstrated that extracellular receptor potentials, not receptor potentials, drive OHC motility and cochlear amplification at high frequencies, and further proposed that the A88V mutation enhances the magnitude of extracellular resting potential in OHCs by blocking the action of the GJ protein to reduce input resistance, which is crucial for the restoration of high-frequency hearing in individuals with the A88V mutation [45].

Additionally, it was proposed A88V mutation of Cx30 not only forms typical, cell-to-cell GJ channels, but also hemichannels that show a propensity to open after insertion in the nonjunctional membrane, which promotes the extracellular release of ATP and other metabolites [44,46]. The extracellular release of ATP may play an important role in preventing the progression of presbycusis.

### 2.3. Mechanism of Connexins in ARHL

ARHL is often accompanied by an imbalance of ROS and a progressive deterioration of metabolism in the inner ear. Dysregulated ROS production has been clearly linked to ARHL in both humans and mice [47,48]. Studies using mouse models have demonstrated a correlation between reduced expression of antioxidative enzymes and accelerated hearing loss, as well as atrophy of SV and degeneration of SGCs [49]. In clinical settings, elevated plasma levels of ROS have been associated with higher pure tone threshold levels [50]. Reduced GJ coupling limits the transfer of nutrients, including glucose, from distant blood vessels to the avascular sensory epithelium in the cochlea. A decrease in glucose supply is a well-known factor that exacerbates ATP exhaustion and increases the levels of ROS.

ROS, as a group of metabolic products including superoxide, hydroxyl radicals, and hydrogen peroxide, can peroxidize membrane lipids of cells and organelles, and damage both DNA and proteins. Modifying diet and increasing antioxidant intake to decrease ROS production in the body has been shown to successfully inhibit the advancement of ARHL in mice [23]. Connexin hemichannels have been observed to facilitate the transport of various molecules up to ∼1 kDa in size, such as ATP, glutamate and glutathione, between the intracellular and extracellular compartments. Diminished GJ coupling hinders the transportation of nutrients, like glucose, from remote blood vessels to the avascular sensory epithelium of the cochlea. A decline in glucose delivery is a recognized factor that worsens ATP depletion and boosts ROS production [51]. It has been suggested that ATP release via hemichannels could trigger inward K^+^ currents, aiding in the entry of K^+^ into supporting cells [52]. This mechanism may assist in reducing the buildup of extracellular K^+^ near hair cells, thereby mitigating the chronic harm caused by elevated K^+^ concentrations to hair cells.

Therefore, ATP is likely an important signaling molecule involved in GJ regulation of presbycusis. It has been suggested that mechanical stimulation can trigger the opening of hemichannels, leading to the release of ATP in response to basilar membrane vibrations induced by acoustic stimulation. ATP, in turn, can modulate the electromotility of OHCs by activating P2 receptors, creating a negative feedback loop that regulates hearing sensitivity and provides protection against cell damage caused by high sound intensity [53]. The propagation of intercellular Ca^2+^ waves within the cochlear epithelium has also been linked to ATP release through hemichannels. Experiments involving the application of ATP in mouse cochlear organotypic cultures demonstrated the activation of purinergic receptors in neighboring cells, resulting in Ca^2+^ release and the spread of Ca^2+^ waves. Interestingly, Ca^2+^ responses did not propagate in cultures lacking Cx26, while they continued to spread in cultures lacking P2X7 receptors or pannexin-1 channels [54]. The role of Ca^2+^ waves in cochlear spontaneous electrical activity prior to auditory maturation is well documented. In particular, it has been suggested that the strength of IP3-mediated Ca^2+^ signaling plays a crucial role in regulating the balance between cell survival, apoptosis, and autophagy [55].

Nrf2 is a crucial transcription factor that regulates the oxidative stress response of cells, playing a key role in maintaining cell redox homeostasis [56]. Nrf2 helps reduce cell damage from oxidative stress and ensures the dynamic balance of systemic redox by inducing and regulating the expression of various antioxidant factors [57]. The dysregulation of the Nrf2/ARE pathway in Cx26 cKO mice is also implicated in the worsening of oxidative stress in the cochlea. In addition, there was a particular role for Cx30 in the organ of Corti in mediating repair responses following hair cell loss [58].

Research has shown that Cx26 mutations can lead to late-onset, progressive hearing loss and congenital deafness [11,18,59]. It was previously thought that reduced EP was the primary cause of hearing loss in cases of Cx26 mutations; however, there is a lack of direct evidence to support this claim. Recent studies propose that congenital deafness stemming from Cx26 mutations is linked to cochlear development and that late-onset hearing loss could be attributed to diminished active cochlear amplification. The frequency discrepancy in delayed hearing loss and reduced active cochlear amplification is particularly pronounced in high-frequency regions, indicating a progressive impact of GJs on cochlear amplification. There is also a view that the *GJB2* mutation (such as p.V37I) leads to a decrease in the efficiency of GJ networks, affecting the transport of molecules such as potassium ions and ATP, resulting in mild and long-term accumulation and progressive hearing loss that gradually occurs with age. These findings suggest that cochlear GJs may play a significant role in ARHL [60].

Previous studies have demonstrated alterations in cochlear blood flow and extravasation in Cx30 KO animal models [61] and highlighted the essential role of Cx30 in maintaining the EP [62]. The EP is essential to hearing because it provides approximately half of the driving force for the mechanoelectrical transduction current in auditory hair cells. Established risk factors for ARHL include elevated oxidative stress and cochlear redox imbalance resulting from connexin downregulation. This downregulation may contribute to SV dysfunction and vascular dysregulation, potentially exacerbating the aging processes within the cochlea [63,64].

Based on these functionally relevant findings, future research could focus on investigating the mechanisms underlying changes in connexin family GJ protein expression during ARHL. This could enhance our understanding of potential treatment options by pinpointing the specific connexin family members and inter-cellular proteins that could be targeted for therapeutic purposes.

## 3. Noise-Induced Hearing Loss (NIHL)

NIHL is a prevalent form of sensorineural hearing impairment that arises from prolonged exposure to high-intensity noise, which can lead to irreversible damage to the cochlea and auditory pathways. As industrialization and urbanization continue to escalate globally, the incidence of NIHL is on the rise, posing significant public health challenges. Approximately 5% of the world’s population is affected by NIHL, making it the second most common cause of sensorineural hearing loss after ARHL [65]. The pathophysiology of NIHL is multifactorial, involving complex interactions between genetic predispositions and environmental factors, particularly occupational noise exposure [66]. Understanding the mechanisms, impacts and preventive strategies for NIHL is crucial for safeguarding auditory health and enhancing the quality of life for those at risk.

### 3.1. Mechanisms of NIHL

The pathogenesis of NIHL is not a singular event but rather the result of multiple interacting pathways, with irreversible damage to cochlear hair cells and their synaptic structures at its core.

Initially, intense noise mechanically injures the organ of Corti, leading to fusion of OHC stereocilia and disruption of IHC ribbon synapses, ultimately triggering apoptosis or necrosis of hair cells [67,68]. This process is accompanied by cochlear microcirculatory dysfunction: noise exposure causes vasospasm and ischemia–reperfusion injury in the inner ear, resulting in a burst of ROS production, peaking 7–10 days after trauma and persisting for over a week [69]. ROS induce lipid peroxidation and DNA damage, triggering the mitochondrial apoptosis pathway and activating the caspase-9/caspase-3 cascade, while also releasing apoptosis-inducing factor and endonuclease G to mediate caspase-independent apoptosis [69,70].

Second, disruption of Ca^2+^ homeostasis is another key mechanism. Noise-induced hyper-depolarization of IHCs leads to excessive Ca^2+^ influx through voltage-gated Ca^2+^ channels, triggering glutamate excitotoxicity [71]. Over-activation of Ca^2+^-permeable AMPA receptors on the postsynaptic membrane causes swelling and vacuolation of the postsynaptic density, manifesting as “hidden hearing loss” [72]. Notably, such synaptic pathology can occur independently of hair cell death, presenting as a reduction in the amplitude of the auditory nerve compound action potential without any elevation of hearing thresholds [72].

Third, inflammatory responses exacerbate the damage. Within two days of noise exposure, bone-marrow-derived monocytes migrate via the CCL2/CCR2 axis to the spiral ligament, differentiate into pro-inflammatory M1 macrophages, and secrete cytokines such as TNF-α and IL-1β [73]. These inflammatory mediators amplify oxidative stress through the NF-κB pathway, forming a positive feedback loop [74]. Recent studies have identified high-mobility group box 1 protein as a damage-associated molecular pattern that aggravates inflammation via the TLR4/NF-κB pathway; inhibition of HMGB1 significantly reduces hair cell loss [75].

Fourth, energy-metabolic failure further worsens the injury. Noise exposure depletes ATP and activates the AMPK pathway. Moderate AMPK activation promotes energy production and is protective, whereas excessive activation drives JNK-mediated apoptosis [76]. Additionally, autophagy clears damaged mitochondria in the early phase of injury, but excessive activation may lead to type II programmed cell death [77].

Furthermore, genetic susceptibility cannot be ignored. OTOF mutations impair ribbon synapse function, rendering individuals more sensitive to noise [78]. Animal models show that *GJB2*-mutant mice exhibit more severe hair cell loss under identical noise exposure [79].

These mechanisms do not act in isolation but form an interconnected network of ROS–inflammation–excitotoxicity interactions, ultimately culminating in the triple hit of synaptic loss, hair cell death, and spiral ganglion neuron degeneration [80].

### 3.2. Effects of Noise Exposure on Connexins and Their Role in NIHL

The impact of noise exposure on cochlear gap-junction proteins is largely confined to the lateral wall. Although some studies report contradictory findings (Table 1), these discrepancies are most likely attributable to variations in noise parameters, sampling time points, and the specific cochlear regions examined. Accumulating evidence demonstrates that the effect of noise exposure on cochlear gap-junction proteins is a multi-step, dynamic, and region-specific process that ultimately disrupts ion homeostasis and intercellular communication, leading to hearing loss. Yamaguchi et al. reported that mice exposed to an 8 kHz octave-band noise at 110 dB SPL for 1 h exhibited a rapid (>50%) reduction in Cx26 protein within the cochlear lateral wall within 4 h, persisting until day 7, whereas the decrease in Cx30 became apparent only on day 7 [81]. Sun et al. observed that in the permanent-threshold-shift mouse model, Cx26 and Cx30 transiently rose at 4 h post-exposure and then precipitously declined, indicating that high-intensity noise initially evokes a “compensatory” synthesis that quickly exceeds cellular protective capacity [82]. Conversely, in the 110 dB temporary-threshold-shift, the central driver of these changes is noise-induced oxidative stress. Within minutes of exposure, ROS surge in the cochlea, leading to massive formation of the lipid-peroxidation product 4-hydroxy-2-nonenal (4-HNE) in the spiral ligament and stria vascularis [81]. 4-HNE is highly electrophilic and forms Michael adducts with lysine residues on Cx26 and Cx30, inducing conformational changes that enhance ubiquitination and subsequent proteasomal or lysosomal degradation. At the transcriptional level, 4-HNE suppresses GJB2 mRNA, so that Cx26 transcript levels drop by 30–50% within 2 h, whereas *GJB6* (Cx30) and GJA1 (Cx43) transcripts are unaffected during this interval [81]. Oxidative stress also activates calpains. Intracellular Ca^2+^ overload rapidly switches on calpain-1 and calpain-2, which cleave the cytoplasmic loops of Cx26 and Cx43, destabilizing gap-junction plaques and removing the proteins from the membrane [83]. Intracochlear administration of the calpain inhibitor PD150606 immediately after noise reduces Cx26 degradation by 70% and restores gap-junction communication, ultimately attenuating the permanent ABR shift by 20–30 dB, demonstrating that calpain-mediated post-translational cleavage is a critical amplification step downstream of 4-HNE [83].

The functional consequence of gap-junction protein loss is collapse of the ion-transport system. Reduced Cx26/30 levels weaken the electrochemical coupling between spiral-ligament fibrocytes and stria-vascularis basal cells, increasing resistance to K^+^ recirculation and lowering the EP from its normal +80 mV to less than +20 mV. Simultaneously, the Na^+^,K^+^-ATPase α1 subunit is down-regulated and its enzymatic activity falls in parallel, further compromising active K^+^ transport. The combined loss of gap-junction channels and pump activity depletes the electrochemical gradient required for cochlear amplification, leading to reduced electromotility of outer hair cells and elevated ABR thresholds. Notably, Na^+^, K^+^-ATPase α1 itself is subject to 4-HNE adduction and subsequent proteasomal degradation, providing a second route by which oxidative stress disrupts ion homeostasis [81].

Interventional studies confirm the centrality of this pathway. Pretreatment with the radical scavenger tempol or the nitric-oxide synthase inhibitor L-NAME, given 30 min before noise, decreases 4-HNE adducts in the spiral ligament by 60–70%, restores Cx26 protein to 50–60% of control levels, normalizes Na^+^,K^+^-ATPase activity, and limits the permanent threshold shift to <10 dB [81]. Similarly, the natural antioxidant Radix astragali attenuates Cx26 down-regulation in the stria vascularis of guinea pigs exposed to impulse noise and reduces the ABR shift by 20–30 dB [87].

Our most recent findings reveal that Cx30-deficient mice exhibit reduced levels of key antioxidant factors such as Nrf2 and HO-1 after noise exposure. This reduction in antioxidant capacity exacerbates the oxidative stress burden, leading to greater cellular damage and impaired recovery [88]. Furthermore, connexin deficiencies disrupt glucose metabolism in the cochlea. Glucose is a critical energy source for cochlear function, and its metabolism is tightly regulated through glycolysis and the tricarboxylic acid cycle. In Cx30-deficient mice, noise exposure results in a significant imbalance in glucose metabolites, characterized by decreased levels of glucose-6-phosphate and cAMP, and increased levels of lactate and NAD+. This shift indicates a reprogramming of glucose metabolism towards anaerobic glycolysis, which produces less ATP compared to aerobic oxidation. The reduced ATP availability further impairs the cochlea’s ability to maintain normal function and repair damage, thereby exacerbating the effects of noise-induced oxidative stress. The combined impact of weakened antioxidant defenses and disrupted glucose metabolism in connexin-deficient cochleae creates a vicious cycle of increased oxidative stress and energy deficiency, ultimately leading to more severe and persistent hearing loss following noise exposure.

Oxidative stress plays a significant role in NIHL. Enhancing the antioxidant defense system to protect GJ function is a potential therapeutic strategy. Studies have shown that antioxidants (e.g., N-acetylcysteine) can reduce oxidative stress damage following noise exposure, protect the expression and function of connexins, and thereby alleviate hearing loss [83].

Moreover, studies have shown that noise exposure reduces the number of synapses in (IHCs and increases intracellular calcium influx—changes that may be associated with the disruption of calcium homeostasis caused by GJ dysfunction [81]. Meanwhile, GJ dysfunction can also impair the nutrient supply and metabolic coupling of IHCs, further exacerbating their functional damage [89].

GJs are implicated in NIHL, as their dysfunction is closely associated with oxidative stress, disruption of ion cycling, and hair cell damage. Further research into the mechanisms of GJ dysfunction in NIHL will not only enhance our understanding of the pathogenesis of NIHL but also provide a theoretical basis for the development of new therapeutic strategies. Future studies should delve deeper into the molecular mechanisms underlying GJ dysfunction and evaluate the potential therapeutic approaches targeting GJ modulation. In the model, both proteins first declined and then recovered within 48 h, demonstrating the reversibility of the lesion.

## 4. The Relationship Between GJs and Cisplatin-Induced Ototoxicity

Cisplatin, a potent chemotherapeutic agent widely employed against various solid tumors, is limited in clinical use due to dose-dependent ototoxicity, which frequently culminates in irreversible sensorineural hearing loss. This ototoxicity preferentially targets outer hair cells, marginal cells of the stria vascularis, and spiral ganglion neurons within the cochlea, thereby significantly constraining its broader therapeutic application. This section explores the role of GJs in cisplatin-induced ototoxicity and discusses potential prevention and treatment strategies.

Cisplatin induces cellular damage and death through several mechanisms: (1) it forms DNA adducts that disrupt replication and transcription, triggering apoptosis; (2) it generates ROS via enzymes such as NADPH oxidase 3, leading to oxidative damage; and (3) it increases pro-inflammatory cytokines, including TNF-α, IL-1β, and IL-6, which activate the MAPK and NF-κB pathways, exacerbating inflammation.

However, in cisplatin ototoxicity, GJs may have a dual role. On one hand, they can mediate the “bystander effect,” where death signals (e.g., ROS and calcium waves) propagate through GJs, causing adjacent cells to die and amplifying the ototoxic damage [90]. For instance, inhibiting GJs with agents like carbenoxolone or knocking down Cx43 has been shown to reduce cisplatin-induced hair cell apoptosis in organotypic cochlear cultures. On the other hand, GJs may also transmit protective signals, such as cAMP, which can activate survival pathways like cAMP/PKA/CREB, reducing cell death [91].

Cisplatin not only directly damages cochlear cells but also affects the expression and function of connexins. Studies have shown that cisplatin decreases the expression of Cx26 and Cx43 in the cochlea, with the reduction being dose- and time-dependent. In cisplatin-treated cochlear cultures, the immunofluorescence intensity of Cx26 and Cx43 decreases, and the opening of GJs is inhibited, reducing intercellular communication efficiency. This weakening of GJ function may further impair cochlear potassium recycling and potential maintenance, exacerbating hearing loss [92].

Regulating GJ activity offers potential strategies for preventing and treating cisplatin ototoxicity. GJ inhibitors, such as carbenoxolone and 18α-glycyrrhetinic acid, have been proven effective in reducing cisplatin-induced hair cell loss and hearing decline in experimental cochlear cultures and animal models by blocking GJs and reducing the spread of death signals [90]. Gene therapy is another promising approach; knocking down or knocking out Cx43 using gene-editing technologies can decrease cisplatin-induced cell death. Overexpressing protective connexins like Cx26 may also protect cochlear cells [92]. Moreover, combining GJ modulation with other otoprotective strategies, such as antioxidants and anti-inflammatory agents, could enhance protective effects by simultaneously inhibiting ROS generation, reducing inflammation, and blocking the spread of intercellular death signals [91].

In conclusion, GJs play a complex dual role in cisplatin ototoxicity, potentially exacerbating cell damage through the “bystander effect” while also being involved in cochlear cell regeneration and repair. Further research into the mechanisms of GJs in cisplatin ototoxicity could lead to the development of effective prevention strategies, reducing the risk of hearing loss in cancer patients undergoing cisplatin chemotherapy and improving their quality of life. Future studies should explore the interactions between GJs and other cellular signaling pathways and work on developing more specific and safer GJ modulators for clinical use.

## 5. Cx31.9 and Ménière’s Disease

Ménière’s disease is an inner ear disorder characterized by endolymphatic hydrops as the primary pathological change, with recurrent episodes of vertigo, hearing loss, tinnitus, and aural fullness as its main clinical manifestations. Although the exact etiology remains unclear, genetic and environmental factors are considered to play significant roles in the disease’s pathogenesis.

Based on the study by Escalera-Balsera et al., gap junctions may contribute to the pathogenesis of familial Meniere’s disease (FMD) through altered intercellular communication in the inner ear [6]. They identified a rare haplotype (TGAGT) in the GJD3 gene, which encodes connexin 31.9 (Cx31.9), segregating with FMD in three unrelated families. This haplotype includes two missense variants, one of which—p.(His175Tyr)—is predicted to disrupt electrostatic interactions between connexins, potentially impairing connexon assembly and homotypic gap junction formation [93,94]. Although functional assays in Xenopus oocytes showed no significant change in hemichannel currents, the structural modeling suggests that the variant may affect channel formation or stability. Immunofluorescence in mice revealed that Gjd3 (the mouse ortholog) is expressed in the cochlea, particularly in the tectorial membrane and beneath inner hair cells, supporting a role for gap junctions in maintaining ionic homeostasis and mechanical signal transmission. These findings suggest that dysregulation of gap junction-mediated signaling, possibly involving potassium cycling or calcium signaling, may underlie inner ear dysfunction in a subset of FMD patients.

## 6. Summary and Outlook

In recent years, the investigation of connexin-based GJ proteins has emerged as a pivotal area of research in understanding the complex pathology underlying acquired hearing loss. As critical components of GJs in the cochlea, connexins—particularly Cx26 and Cx30—facilitate intercellular communication essential for maintaining cochlear homeostasis and auditory function, especially via potassium recycling and ATP release. However, their role should be regarded as one node within the pathological network of the cochlea, rather than a singular driving force [95,96]. Current evidence indicates that aging, noise overexposure, or cisplatin can reduce Cx26/Cx30 expression or alter channel gating, concurrently with impaired K^+^ recycling, exaggerated oxidative stress, metabolic imbalance, and disturbed Ca^2+^ homeostasis.

For instance, exposure to loud noises leads to significant connexin degradation, which coincides with calpain activation and 4-HNE adduction; this parallels impaired potassium recycling and ATP release vital for hair cell function, though their relative impact can only be partially dissected through interventional studies. Similarly, ototoxic medications like cisplatin induce alterations in connexin expression, where gap junctions may propagate either pro-death signals or cytoprotective cues—with the net outcome context-dependent on drug dose, exposure time, and concomitant injury pathways. In ARHL, connexin down-regulation (e.g., decreased Cx30/Cx43) correlates with functional declines in auditory brainstem responses and distortion product otoacoustic emissions, and appears mutually reinforcing with oxidative stress, though temporal causality remains equivocal. Even in Ménière’s disease, while co-segregation of Cx31.9 variants with the clinical phenotype points to association (not causation), the pathogenic sequence awaits functional validation. Notably, these connexin alterations largely parallel—rather than precede—hair-cell loss or spiral-ganglion neuron degeneration, leaving an independent and decisive contribution unestablished [97,98].

Studies using animal models further highlight this complexity: the absence of Cx30 exacerbates age-related cochlear damage and heightens susceptibility to auditory decline, suggesting connexin dysfunction may serve as an early biomarker. Additionally, genetic predisposition (e.g., *GJB2* variants encoding Cx26) interacts with environmental factors to produce variable phenotypic expressions of hearing loss, underscoring the multifactorial nature of connexin-related pathologies.

Collectively, connexins contribute to cochlear microenvironmental stability, yet their significance must be appraised cautiously within a multi-factorial interactive framework. While their downregulation is closely linked to the pathophysiological mechanisms of hearing impairment, therapeutic strategies aimed at restoring connexin function require careful consideration of concurrent injury pathways. Future research should focus on elucidating the specific molecular pathways involved in connexin dysregulation and exploring interventions to enhance their expression or function, ultimately improving auditory outcomes for at-risk individuals.

## Figures and Tables

**Table 1:** Studies on the effects of noise exposure on Cx expression in the spiral ligament (SL), basilar membrane (BM), stria vascularis (SV) and whole cochlea.

Noise			Region	Cx26	Cx30	Animal	Ref.
dB SPL	Type	Duration					
115	White noise	48 h	SL	↑	—	Wistar rats	[84]
110	Octave band, 8 kHz	1 h	SL	↓	—	Std-ddY mice	[85]
100	Narrow band, 4 kHz	8 h/d × 3 d	BM, SV, SL	↓	↓	Kunming mice	[86]
176	Impulse noise (rifle shots)	15 × 1 s	SV	↓	—	Albino guinea pigs	[87]
120	Broadband	2–4 h	Whole cochlea	↑ at 4 h → ↓ by day 7	Mirrors Cx26	C57BL/6 mice	[82]
110		1 h		↓ at 4 h → recovery by day 2	Mirrors Cx26		

## Data Availability

Data are derived from cited published studies and accessible via the references listed in this review.

## Abbreviations

The following abbreviations are used in this manuscript:

4-HNE	4-hydroxy-2-nonenal
ABR	Auditory Brainstem Response
AMPK	AMP-activated protein kinase
ARHL	Age-Related Hearing Loss
ATP	Adenosine Triphosphate
BM	Basilar Membrane
CCL2	Chemokine (C-C motif) Ligand 2
CCR2	C-C Chemokine Receptor type 2
CD-1	a mouse strain (originally “Carworth Farms”)
CM	Cochlear Microphonic
CREB	cAMP Response Element-Binding protein
Cx26	Connexin-26
Cx30	Connexin-30
Cx30.2	Connexin-30.2 (mouse ortholog of human Cx31.9)
Cx31.9	Connexin-31.9
cKO	conditional Knockout
EC	extracellular loops
EP	Endocochlear Potential
FMD	familial Ménière’s Disease
GJ	Gap Junction
*GJB2*	Gap Junction β-2 gene (encodes Cx26)
*GJB6*	Gap Junction β-6 gene (encodes Cx30)
*GJD3*	Gap Junction δ-3 gene (encodes Cx31.9 in humans)
HED	Hidrotic Ectodermal Dysplasia (Clouston syndrome)
HMGB1	High-Mobility Group Box 1 protein
HO-1	Heme Oxygenase-1
IHC	Inner Hair Cell
IL-1β	Interleukin-1 beta
IL-6	Interleukin-6
IP3	Inositol 1,4,5-trisphosphate
JNK	c-Jun N-terminal kinase
K^+^	Potassium ion
MAPK	Mitogen-Activated Protein Kinase
MIM	Mendelian Inheritance in Man (database identifier)
Na^+^	Sodium ion
NAD^+^	Nicotinamide Adenine Dinucleotide (oxidized form)
NADPH	Nicotinamide Adenine Dinucleotide Phosphate (reduced form)
NF-κB	Nuclear Factor kappa-light-chain-enhancer of activated B cells
NIHL	Noise-Induced Hearing Loss
Nrf2	Nuclear factor erythroid 2-related factor 2
OHC	Outer Hair Cell
OTOF	Otoferlin gene
P2 receptors	Purinergic receptors activated by ATP and related nucleotides
P5	Postnatal day 5
PD150606	a selective calpain inhibitor (N-(4-fluorophenyl)sulfonyl-L-valyl-L-leucyl-4-aminobutyric acid)
PKA	Protein Kinase A
ROS	Reactive Oxygen Species
SGN	Spiral Ganglion Neuron
SL	Spiral Ligament
SLFs	Spiral Ligament Fibrocytes
SV	Stria Vascularis
TNF-α	Tumor Necrosis Factor-alpha
TLR4	Toll-like receptor 4
TM	transmembrane segment
